# Risk factors and therapeutic measures for postoperative complications associated with esophagectomy

**DOI:** 10.1016/j.amsu.2020.05.011

**Published:** 2020-05-23

**Authors:** Mojtaba Ahmadinejad, Ali Soltanian, Leila Haji Maghsoudi

**Affiliations:** Department of General Surgery, Faculty of Medicine, Alborz University of Medical Sciences, Karaj, Iran

**Keywords:** Esophageal cancer, Chemoradiotherapy, Surgery, Risk factor, Complications

## Abstract

Esophageal cancer is one of the most common cancers associated with the high mortality rate. Timely diagnosis and treatment are important to manage the disease and prevent comorbidities. Surgical resection of the tumor and lymph nodes is usually practiced either with or without chemo or chemoradiotherapy. Despite advancements in surgical methods and skills, complex nature of the esophagus and invasiveness of the surgery can lead to serious complications in these patients. In order to predict postoperative outcomes, preoperative examination of the patients, in addition to risk factors, should be conducted. **Conclusion**: Lastly, early detection of adverse postoperative events may help faster recovery, reduce hospital stay and prevent other morbidities.

## Highlights

• Esophageal cancer (EC) is forth most widespread cause of mortality.
• Esophagus is a complex organ with limited abilities of self-repair.
• early detection of adverse postoperative events may help faster recovery, reduce hospital stay and prevent other morbidities.

## 1 Introduction

**Figure 1:**
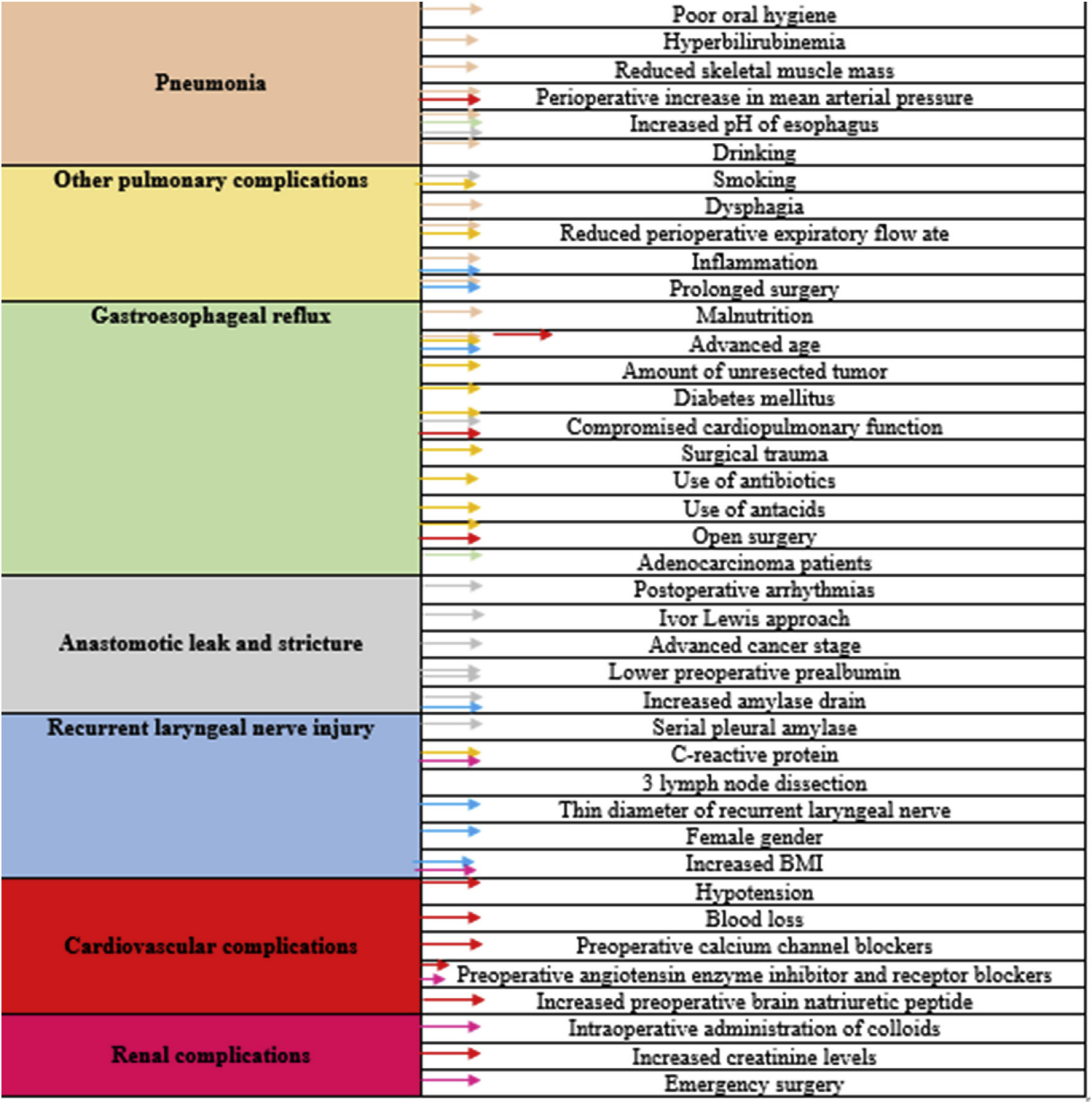
Highlights some of the major preoperative and intraoperative factors (on the right) that can lead to the complications (on the left).

Esophageal cancer (EC) is the eight commonest cancer reported and is forth most widespread cause of mortality worldwide where, esophageal squamous cell carcinoma having the highest incidence [1]. Barrett's esophagus, due to gastroesophageal reflux disease (GERD), is associated with 30–40% risk of esophageal adenocarcinoma. Eventual shift of squamous epithelium to columnar epithelium, known as metaplasia, is seen as a result of the acidic environment in the esophagus [2]. Initially, the cancer is presented in the mid-third of the thoracic esophagus where these lesions advance into polyps and tumor, leading to the blockage of the lumen and invading other layers of the esophagus [3]. Additionally, risk factors such as; smoking, alcohol abuse, obesity, gastroesophageal reflux disease, viral infection, poverty, esophageal achalasia and genetic and epigenetic factors contribute chiefly to the onset of EC [4,5]. Several markers are used for the detection of esophageal cancer cell lines such as; CD44, aldehyde dehydrogenase, p75NTR, CD 90 (Thy-1), NANOG, Podoplanin, CD133, SALL4 and COX2 [1]. Early detection, diagnosis and treatment are possible for esophageal cancer, owing to the innovations in the medicine, however, 5-year survival rate of these patients is limited to 20% only [5]. (see Fig. 1)

Endoscopic resection, esophagectomy, is a commonly performed surgery for resectable esophageal tumors [6]. Depending on the physical health of the patients and the stage of tumor therapeutic intervention is chosen. In Barrett-esophagus and early stage of cancer endoscopy or surgery is performed whereas, in advanced stages, with or without surgery, chemo or chemoradiotherapy is performed preoperatively [7].

Surgery usually comprises of lymph node dissection and esophageal reconstruction. It is an extremely invasive surgery; therefore, great number of complications are associated with its outcomes [8]. Recently, thoracoscopic methods became integrated with minimally invasive (MI) laparoscopic approaches to achieve better advantages. Integration of 3D cameras have also allowed surgeons to view histological and micro-anatomical organizations during the surgery [9]. Single-port mediastinoscopy using transmediastinal and cervical approaches has been performed in a recent few years [10,11] that can lead to reduction in perioperative complications [11]. Advancements in surgical techniques are likely to reduce the frequency of postoperative mortality and morbidities [12]. Esophageal cancer surgery is considered among the most invasive cancer surgeries and is therefore associated with 60–80% adverse postoperative events and corresponding reduced overall survival rate [13,14].

Postoperative complications and morbidities are associated with some common risk factors such as; smoking and alcohol consumption, advanced age, increased BMI, malnutrition, preoperative heart problem and McKeown Esophagectomy [15].

This review is designed to summarize some of the most frequent complications reported after esophageal cancer surgery (esophagectomy), associated risk factors and therapeutic interventions that can treat or prevent these events.

## 2 Pulmonary complications

Pulmonary complications account for 20% of esophagectomy postoperative outcomes. Amount of unresected tumor, advanced age, diabetes mellitus, compromised pulmonary function, history of chronic obstructive disease (COPD), smoking, use of alcohol, location of the tumor, and surgical trauma are some of the risk factors that can lead to the increased incidence of pulmonary adverse events [[16], [17], [18]].

Postoperative pneumonia (PP) is one of the most fatal and severe postoperative complications marked after esophagectomy [19]. Aspiration of oropharyngeal fluid with the bacterial agents that gets attached to the mucosa of lower respiratory tract, can lead to PP [20]. Some of the commonly reported pathogenic microbes involved in PP include *pseudomonas aeruginosa, klebsiella pneumoniae,* methicillin resistant *staphylococcus aureus* and xanthomas maltophilia [21]. Prolonged operation time, dysphagia, drinking and smoking are significantly related with the incidence of pneumonia.

Several studies have suggested that maintenance of oral hygiene before and after surgery by brushing teeth and tongue, breathing training and halting smoking can reduce the incidence of pneumonia [18]. Oral bacteria have been considered as one of the major causes of postoperative pneumonia following esophageal surgeries [22].

Additionally, perioperative increase in mean arterial pressure and pH and low preoperative peak expiratory flow can also predict postoperative pneumonia [23,24]. Asaka, Shimakawa [22] studied the relation between systemic inflammatory response syndrome (SIRS) and pneumonia in esophagectomy patients. Authors reported significant correlation between SIRS and PP. Overproduction of inflammatory cytokines are likely to activate adhesion molecules, that assist the attachment of bacteria to the mucosa. These cytokines impair the integrity of epithelium, thus assisting colonization of the bacteria [22]. Interestingly single nucleotide polymorphism of IL-10 (−819 T/T) is associated with greater incidence of PP and decreased levels of postoperative IL-10 [25]. Moreover, postoperative hyperbilirubinemia needs to be monitored since it is also considered as an additional factor to the complications like pneumonia [26]. Geriatric patients presenting malnutrition and reduced skeletal muscle mass (measured by psoas muscle index) have higher risk of acquiring PP, and may reduce overall survival rate [27]. Bronchial bleeding is a scarcely reported complication associated with PP following esophageal cancer surgery [28].

Sato, Motoyama [29] presented that approximately 29–39% of patients undergoing esophagectomy are presented with poor oral hygiene and mild to poor periodontitis [30]. Among these patients, 13% are at the risk of acquiring pneumonia where, preoperative dental examinations are associated with the decrease in the incidence. Pneumonia is also associated with the decrease in overall survival rate, following the surgery [31]. Moreover, preoperative neoadjuvant chemotherapy can significantly decrease the frequency of pneumonia [32]. Liu, Lian [33] provided a therapeutic method known as "bundle therapy" to treat pneumonia after cervical esophagectomy. Tracheostomy with ventricular assistance, hemodynamic support, enteral administration of food via tube and usage of antibiotics and expectorants can effectively treat complicated pneumonia and reduce the risks of other complications.

Chronic obstructive pulmonary disease (COPD), sarcopenia (in geriatric patients) and postoperative delirium have been also reported to increase the risk of these pulmonary complication following esophagectomy [34,35].

Pulmonary infections are reported in nearly all type of surgical methods; including minimally invasive procedures. Liu, Peng [36] reported that age, usage of antacids and antibiotics, diabetes, cardiovascular and pulmonary disease and increased duration of hospitalization are significantly important factors associated with postoperative pulmonary infections. Nonetheless, in comparison with open esophagectomy, one year follow up results have shown that minimally invasive procedures are associated with lesser pulmonary complications like decrease in forced expiratory volume and volume capacity [37]. Similar outcomes are reported in response to neoadjuvant therapy followed by MI esophagectomy using Ivor Lewis method [38].

Chylothorax is among the rarest complications seen after esophagectomy, that is characterized by the accumulation of fluid (chyle) in the pleural cavity due to the surgical trauma [39]. Despite the incidence of chylothorax is very low (0.5–3%), severity of the complication can be fatal. Additionally, it can lead to hypovolemia, loss of protein, nutrients and important immunological molecules [40]. Location of the tumor, incomplete response of patient to neoadjuvant chemoradiation and challenging mediastinal dissection are common perioperative risk factors associated with the greater incidence of chylothorax where transabdominal mass ligation can reduce the risk [41]. To it, perioperative prophylactic ligation of thoracic duct is also known to manage the complication [42,43].

Wang, Chen [44] reviewed that administration of a neutrophil elastase inhibitor (sivelestat) in patients during esophagus surgery reduces the incidence of postoperative need of mechanical ventilation and acute lung injury. However, its effects on pneumonia, duration of hospitalization and other associated complications might require more studies.

## 3 Gastroesophageal reflux (GER)

GER is commonly reported in patients after esophagectomy. Studies have reported that esophageal acid reflux can increase up to 28% followed by heartburn and regurgitation after the surgery. Disruption of antireflux mechanism by the lower esophageal sphincter and associated anatomical structures during the surgery can lead to reflux. It is also significantly associated with other complications like anastomotic stricture and PP. Increased esophageal pH, pathologic bolus and acid exposure are seen in these patients. Proton pump inhibitors can be used for the treatment of GER [45]. In a recent study, Fuchs, Schmidt [46] demonstrated that patients who underwent adenocarcinoma and squamous cell esophageal carcinoma surgeries had increased reflux-dependent mucosal damage [47], 5 years following the operation whereas, Barrett's esophagus was reported in 20% patients. These findings were common in adenocarcinoma patients. Side overlap with fundoplication has been recently introduced as a surgical technique, that can be performed laparoscopically and is likely to reduce the incidence of postoperative reflux [48]. Reconstruction of gastric tube, rather than traditional anastomosis of esophagus to the unresected gastric parts, can also reduce the frequency of GER in patients undergoing esophagectomy for adenocarcinoma [49].

Patients presenting with postoperative gastric reflux more than once a week have reduced overall health status and are likely to have greater incidence of fatigue, nausea, sleeplessness, vomiting and breathing problems [50].

## 4 Anastomotic leakage (AL) and anastomotic stenosis/stricture (AS)

AL is one of the commonest postoperative complications associated with wide range of surgeries. However, contributing risk factors and therapeutic interventions differ, owing to the type of pathology and surgery performed. For esophagectomy, decrease in perioperative pH [23], smoking, postoperative arrhythmias and other adverse cardiac events [51], Ivor Lewis approach and advance stage of cancer [52,53] are factors leading to anastomotic leakage and stricture. In a recent study, Gao, Xu [54] identified that lower preoperative serum levels of prealbumin and amylase concentration in the drainage are characterized by the risk of anastomotic leakage in McKeown method of minimally invasive esophagectomy and early detection using these parameters is likely to reduce severity and further complications. Similarly, postoperative serial pleural amylase and c-reactive proteins levels are also the indications of anastomotic leak [55,56].

Furthermore, Collard anastomosis is identified with reduced risk of anastomotic stenosis in variant with the hand sewn ones [57]. Studies have shown that anastomotic leak contributes chiefly to postoperative mortality. To it, it can prolong the duration of hospitalization, delay oral feeding might add to the risk of reoperation, in case of sepsis [58].

Double and triple layered sutures in MI surgery and the implantation of endoluminal stent [59] are some of the effective methods proposed to reduce the incidence of anastomotic leakage and stenosis [60,61]. To it, ischemic preconditioning of the stomach may inflict these outcomes too [62]. Early postoperative endoscopy could be exploited for the detection and management of these conditions, followed by timely therapeutic measurements [63].

## 5 Recurrent laryngeal nerve injury

Surgical trauma like stretch, compression or thermal shock to laryngeal nerve can lead to nerve palsy relatively up to 60%. However, discrepancies in these finding are relative to the technical aspects of the surgery and the type and size of the tumor to be resected. Recurrent laryngeal nerve (RLN) is a branch of the vagus nerve which innervates the esophageal muscles where it contributes to the process of swallowing [[64], [65], [66]]. Koyanagi, Igaki [67] reported that in the dissection of three lymph nodes during esophagectomy, approximately 29% patients had RLN injury. Unilateral RLN palsy is characterized by vocal cord paralysis, whereas, among all the cases of RLN palsy, incidence of respiratory complications, anastomotic leak and longer hospital stay was significantly greater, in comparison with the patients in whom RLMN reconstruction was not performed. Prolonged operating time and old age were the risk factors of the nerve injury in this study. In a recent study, it is reported that thin diameter of RLN, female gender and increased BMI are the significant risk factor for RLN-mediated left vocal cord paralysis [68].Other complications associated with RLN paralysis include; pneumonia, acute respiratory distress syndrome, breathlessness during speech, strain during cough, atelectasis and suffocation. Scholtemeijer, Seesing [64] in their study revealed that diabetic patients with advanced age are more prone to acquire pulmonary complication, following RLN paralysis. Within 6 months, half of the affected population showed complete recovery whereas, patients who did not recover were provided with surgical intervention where, 1 patient failed to show recovery at all.

Meanwhile, 32% of unilateral RLN palsy cases are asymptomatic and undiagnosed unilateral injury can lead to bilateral paralysis therefore, laparoscopic screening should be conducted by the surgeons, keeping risk factors in consideration [69]. Furthermore, intraoperative nerve monitoring by the stimulation of vagus nerve, during minimally invasive procedures can also help surgeons to detect nerve injury, decreasing the risks of future complications [70,71].

Gene therapy has also been suggested as an alternative method to hasten nerve repair by injection neurotrophic and growth factors [72].

## 6 Cardiovascular complications

Intra and post-operative adverse cardiac events, in non-cardiac disease surgeries are one of the most common causes deaths. Improved technical aspects of the surgery and intraoperative monitoring of cardiovascular activity can reduce the incidence of these events. Esophagectomy is associated with the greatest odds of cardiac arrest, deep vein thrombosis (DVT) and myocardial infraction, among various other types of abdominal surgeries [73,74]. Hypotension, as a result of intraoperative fluid shift is also reported in some cases [75]. Minimally invasive procedure is superior to open esophagectomy in regards with adverse cardiovascular events [76,77].

The surgical apgar scoring system can be exploited to successfully measure intraoperative adverse cardiac events such as; hypertension, blood loss, decreased arterial pressure and heart rate and predict the risk of acquiring short and long term postoperative complications such as, pneumonia and anastomotic leakage [78,79].

The incidence of DVT following esophagectomy is reported as 2.9–13.7% [80].

A study by Yoshida, Baba [80] indicated that prophylactic treatment enoxaparin is likely to reduce the incidence of DVT after esophagectomy. A survey of practice pattern by thoracic surgeon reported that chemoprophylaxis with low-dose unfractionated heparin or low-molecular-weight heparin before esophagectomy is essential for DVT [81].

In a retrospective review, Colwell, Encarnacion [82] reported that 32.4% patients who underwent transcervical esophagectomy developed postoperative atrial fibrillation (AF) which was characterized by prolonged ICU and hospital stay. Another retrospective study recruiting 121 patients reported the incidence of AF as 31.4% and advanced age, chemoradiation and male gender were known risk factors. Preoperative intake of amiodarone is beneficial against the risk of AF after the surgery. Minimally invasive and open esophagectomy have similar incidence of AF, reported in another retrospective study [83]. However, transthoracic approach in advanced-age patients with the history of cardiopulmonary diseases increases the risk of the development of AF, adding to days of hospitalization and other complications [84]. In a study by Ojima, Iwahashi [85], following transthoracic esophagectomy, atrial fibrillation was reported in 9.2% of the patients where antiarrhythmic therapy using landiolol hydrochloride was effective in 63.2% patients.

Preoperative usage of calcium channel blockers, angiotensin converting enzyme inhibitors and blockers of angiotensin receptor can reduce the risk of AF, leading to decrease in overall survival rate and subsequent mortality [86]. In a randomized clinical study, prophylactic use of landiolol hydrochloride in patients undergoing transthoracic esophagectomy was marked with the reduced frequency of AF, with the suppression in the heart rate and levels of IL-6 [87].

## 7 Acute kidney injury

Renal complications are reported in 3% cases following gastroesophageal surgeries. Risk factors such as; increased BMI, use of angiotensin enzyme inhibitors and receptors blockers, intraoperative administration of colloids, increased postoperative C-reactive protein, preoperative hypertension and diabetes mellitus and increased creatinine levels are factors associated with the increased incidence of acute kidney injury following esophageal cancer surgery [88,89].Whereas, administration of IV dexamethasone and ketorolac can reduce this risk [13,90]. Japanese herbal medicine (Daikenchuto, TJ-100) has been reported as a therapeutically effective compound after esophagectomy. Its efficacy is characterized by decrease in postoperative c-reactive protein levels thereby, repressing the inflammatory response [91,92]. Lin, Huang [93] reported that patients undergoing major surgical procedures like esophagus cancer surgery are prone to acquire acute kidney injury-requiring dialysis and this complication is associated with the increased mortality, hospital stay and other comorbidities [94]. Emergency esophagectomy in response to perforation of esophagus followed by chemoradiotherapy is also characterized with the increased incidence of acute renal failure [95]. In a recent retrospective reviewed study, 4.7% of patients diagnosed with squamous cell carcinoma who underwent minimally invasive esophagectomy using McKeown approach developed acute kidney injury (AKI) [96].

Wang, Wang [97] reported that among 2094 patients who underwent esophageal cancer surgery, AKI was seen in 2.4% patients. Sugasawa, Hayashida [98] reported that reduction is stroke volume index after esophagectomy can be a risk factor for AKI. Other risk factors include obesity, cardiovascular comorbidities, increased preoperative creatinine concentration [99], use of angiotensin-converting enzyme inhibitors or angiotensin-receptor blockers, preoperative albumin levels, postoperative c-reactive protein, colloid infusion during surgery [100], transthoracic approach and chemotherapy and chemoradiation therapy [101].

Additionally, preoperative renal dysfunctions can lead to the other surgical complications such as; anastomotic leakage [[102], [103], [104]].

## 8 Conclusion

Esophagus is a complex organ with limited abilities of self-repair. Esophageal malignancies suffer from limited treatment options. Surgical interventions and chemoradiotherapy are most widely practiced in this aspect. However, invasiveness of the surgical procedure is associated with the number of peri and post-operative complications including, mortality. Efficient management of these adverse event contributes to the success of overall therapeutic procedure.

Patients' history, detailed examination and preoperative preventive measures can improve surgical outcomes. Early diagnosis of the cancer can be treated by lesser non-invasive therapies such as endoscopic radiofrequency ablation for Barrett's esophagus and endomucosal resection for nodular conditions. Moreover, tissue engineering has opened great diversity of therapeutic alternatives such as; stem cell therapy, bio-scaffolds and biomaterial, which are under animal-based, preclinical and clinical investigations for esophageal reconstruction [5]. A number of animal studies have reported successful outcomes in this regard, such as the use of tubelized acellular matrix autologous skeletal myoblasts, enclosed by human amniotic membrane and seeded with autologous epithelial cells for esophageal stenosis and extracellular matrix scaffolds made of porcine urinary bladder extracellular matrix [105,106] Multidisciplinary approach is available of esophageal cancer, nonetheless, detailed studies regarding side-effects of these therapies can improve the outcomes of the procedures and hasten recovery.

### 8.1 Limitations

Our study is limited to a narrative review, therefore, doesn't provide statistical outcomes regarding the complications and the risk factors. Additionally, the study is focused on commonly reported complications. Rare cases, which cannot be foregone, are not discussed. Detailed meta-analysis and systematic reviews can give us better conclusion.

### Provenance and peer review

Not commissioned, externally peer reviewed.

## Ethical Approval

All procedures performed in this study involving human participants were in accordance with the ethical standards of the institutional and/or national research committee and with the 1964 Helsinki Declaration and its later amendments or comparable ethical standards.

## Funding

None.

## Author contribution

Dr. Mojtaba Ahmadinejad: conceptualized and designed the study, drafted the initial manuscript, and reviewed and revised the manuscript.

Dr. Ali Soltanian: Designed the data collection instruments, collected data, carried out the initial analyses, and reviewed and revised the manuscript.

Dr. Leila Haji Maghsoudi: Coordinated and supervised data collection, and critically reviewed the manuscript for important intellectual content.

## Guarantor

The Guarantor is the one or more people who accept full responsibility for the work and/or the conduct of the study, had access to the data, and controlled the decision to publish.

## Human and animal rights

No animals were used in this research. All human research procedures followed were in accordance with the ethical standards of the committee responsible for human experimentation (institutional and national), and with the Helsinki Declaration of 1975, as revised in 2013. This study was approved by the Research Ethics Board of Alborz University of Medical Sciences.

## Consent for publication

Informed consent was obtained from each participant.

## Availability of data and materials

All relevant data and materials are provided with in manuscript.

## Declaration of competing interest

The authors deny any conflict of interest in any terms or by any means during the study.All the fees provided by research center fund and deployed accordingly.
